# Developing a Multilayer Framework for Integrating Oral Health into General Health: A Scoping Review from Oral Healthcare Workers’ Perspectives

**DOI:** 10.3390/healthcare14070918

**Published:** 2026-04-01

**Authors:** Peivand Bastani, Manori Dhanapriyanka, Hongmei Xie, Ratika Kumar, Diep Hong Ha

**Affiliations:** 1 College of Business, Creative Arts, Law and Social Sciences, Flinders University, Centre for Social Impact, Adelaide, SA 5042, Australia; 2 Oral Health Centre, School of Dentistry, Faculty of Health and Behavioural Sciences, The University of Queensland, Brisbane, QLD 4006, Australia

**Keywords:** oral health, general health, primary healthcare, integration, oral healthcare professional

## Abstract

**Highlights:**

**What are the main findings?**

- Five principal integration domains were identified in which oral health professionals can play a substantive role within general health settings: (1) chronic disease management, encompassing screening, counseling, and referral; (2) emergency care management; (3) integration of electronic health records (EHRs); (4) interprofessional education across undergraduate, postgraduate, and continuing professional development levels; and (5) telehealth, including tele-dentistry.

**What are the implications of the main findings?**

- Advancing integration will require coordinated governance reform, workforce development, digital infrastructure strengthening, and community engagement.

**Abstract:**

**Background**: Oral healthcare workers play a pivotal role in exploring the significant potential of integrating oral healthcare with overall health within a healthcare system. This review aims to identify the main barriers and facilitators to integrating oral health into primary and general healthcare from the perspectives of oral healthcare professionals. **Methods**: The study adhered to the Arksey and O’Malley methodological framework for scoping reviews. Five main databases were systematically searched, namely Web of Science, PubMed, Scopus, ProQuest, and Embase, spanning from 1 January 2000 to 31 December 2024. The Rainbow Model served as the framework for content analysis, organizing the advantages, disadvantages, barriers, and facilitators into micro, meso, and macro levels. **Results**: Five integration domains were identified across macro, meso, and micro levels, illustrating how oral health can be systematically embedded within general health through the utilization of oral healthcare professionals. These domains encompassed chronic disease management (screening, counseling, and referral), emergency management, electronic health records, interprofessional education, and tele-dentistry, highlighting policy, organizational, and workforce levers for strengthening care integration, enhancing system efficiency, and improving access and equity. **Conclusions**: This scoping review synthesizes five integration domains and four cross-cutting strategic directions for embedding oral health within broader healthcare systems. By conceptualizing integration across macro, meso, and micro levels, the study provides a structured framework that may serve as a reference for policymakers, educators, and health service leaders. The findings highlight potential enablers, such as coordinated governance, workforce development, digital infrastructure, and community engagement, which could support integration.

## 1. Introduction

The concept of integration has been discussed as a multidisciplinary approach in healthcare systems to improve the effectiveness, quality, access, and continuity of healthcare services [1]. “Integration” was developed and defined by Grone and Garcia-Barbero (2001) as a function of bringing all the inputs, delivery, management, and organizations related to diagnosis, treatment, care, rehabilitation, and health promotion together [2] to improve the overall health in one place. This integrated care approach has been introduced in primary healthcare systems to provide and allow for global access to different categories of healthcare services [3].

Oral health has long been well-recognized as an integral particle of general health. It is one of the disciplines where attempts have been made to integrate it with primary healthcare. Despite all attempts in this area, the dental field is still a separate health sector, and dental caries is still among the most common diseases that can affect 60–90% of children and most adults worldwide [4]. The existing high prevalence of this disease is strong evidence that implies oral diseases are a neglected issue, and they are rarely considered a priority in health policy [5].

Historically, in 2009, the concept of integrating oral healthcare into primary healthcare services was advocated at the World Health Organization’s 7th global conference [6]. However, the different nature of oral health services, including the costs, has caused low-income, uninsured families and vulnerable groups to become the most frequently cited when it comes to unmet dental care needs [7]. As a result, the American Academy of Family Physicians has moved toward the integration of oral health into primary healthcare by creating a suitable framework. The framework consists of multidisciplinary collaborative including assessing oral health risk factors and symptoms, conducting oral health evaluations, implementing preventive interventions, and providing oral health education [8]. In another American case study, a pilot collaboration was undertaken to develop projects addressing integrated oral health and various chronic diseases, such as obesity, diabetes, heart disease, stroke, and tobacco use across six states [9]. The results of this case study demonstrate the effectiveness of promoting collaboration within state health departments. Through this collaborative effort, dental and medical clinicians received training, patients were educated on clinical prevention measures, referral systems were established, and impactful media campaigns were launched to disseminate crucial information [9].

Despite these movements, there are still many obstacles to integrating oral health into general health. For instance, Bernstein et al. (2016) [10] have shown that there are many barriers to integrating oral healthcare into pediatric medical practice at health centers. Such an integrated system will require adapting delivery systems to support multidisciplinary collaboration by applying various strategies including facilitating oral health data collection, extraction and documentation, regular feedback, training of pediatric health staff in preventive oral health, increased funding for oral health, and a focus on connecting pediatric and dental efforts [10]. Harnagea et al. (2017) have also summarized the barriers to the integration of oral health into primary healthcare as follows: lack of political leadership and healthcare policies, lack of competencies and education, lack of continuity of care and services, undefined oral healthcare needs of patients, and many challenges related to the implementation of such an integration [3].

Considering the importance of an integrated health system and potential barriers, it seems that there is a strong need for advocacy in using a population health approach to integrate oral health within primary healthcare. The necessity of integration becomes more highlighted considering that oral health can be related to some specific general conditions such as heart disease, diabetes, and pre-term low-weight babies [11]. Such evidence simply emphasizes that to provide whole-person, integrated, patient-centered, comprehensive care, a healthcare system must connect the mouth to the rest of the body, as well as oral health to general and primary health [12].

From the financial and good utilization of the oral services perspective, the results of a survey in five developed countries including the UK, the USA, Canada, Australia, and New Zealand have clearly emphasized that the integration of oral health within primary healthcare cannot happen unless attempts are made to bring oral healthcare and primary healthcare under one roof and oral health services are provided as a section of the comprehensive package of healthcare [13]. The integration mentioned not only demands public accountability in financing but also necessitates a shift toward providing oral health services with universal access to preventive, promotive, and restorative care.

In this regard, the role of oral healthcare professionals is as significant as formulating and regulating policies. Christian et al. (2022) have mentioned that preventive oral health services should be delivered by primary care professionals in the primary care setting, where integration can happen via training healthcare workers and policy changes [14]. According to the findings of this scoping review, numerous integration strategies have been identified, primarily focusing on enhancing referral pathways, streamlining documentation procedures, optimizing operational efficacy, augmenting the healthcare workforce dedicated to oral health services, and implementing preventive interventions targeting dental caries [14].

Regarding the crucial role of healthcare professionals, there is evident potential to investigate how a healthcare system can incorporate oral health into general health by leveraging oral healthcare professionals. The integration scenario is not only designed to cope with the workforce shortage, or the manifestation of early indicators of systemic diseases through the oral cavity, but it also promotes a willingness and familiarity from both sides of the profession to collaborate in a unified team. This scoping review aims to explore the primary barriers and facilitators of integrating oral health with primary healthcare/general healthcare from the perspectives of oral healthcare professionals. Additionally, it seeks to examine the key advantages and disadvantages of integrating oral health with primary healthcare/general health through the involvement of oral healthcare professionals. The ultimate outcome of this study is a conceptual framework for integrating oral health within primary and general healthcare systems through the effective involvement of oral healthcare professionals.

## 2. Materials and Methods

This study was conducted in 2022 and followed the Arksey and O’Malley methodological framework for scoping reviews [15], encompassing six key stages: identifying the research question, searching for relevant studies, selecting studies, charting and collating data, summarizing and reporting results, and consulting with stakeholders to inform the review [optional]. This study was reported using the Preferred Reporting Items for Systematic Reviews and Meta-Analysis extension for Scoping reviews [PRISMA-ScR] [16]. To guide this review, the Rainbow Model was used as the conceptual framework [17] because it provides a comprehensive framework for understanding integrated care across multiple levels, including clinical, professional, organizational, and system integration. This multidimensional perspective aligns well with the aims of our study, which explores integration of oral healthcare professionals within primary and general healthcare systems.

A review protocol was entered into the Open Science Framework (registration: https://doi.org/10.17605/OSF.IO/AFD3Z).

### 2.1. Identifying the Research Question

The research question addressed in this review was “What are the barriers, facilitators, advantages, and disadvantages of integrating oral health with primary healthcare/general health in both Australia and worldwide, using oral healthcare team professionals?”.

### 2.2. Identifying Relevant Studies

The search strategy was developed in collaboration with a University of Queensland librarian, and an electronic search was conducted across five primary databases, namely Web of Science, PubMed, Scopus, ProQuest, and Embase, covering the period from 1 January 2000 to 31 December 2024. After removing duplicate studies, all remaining records were imported into the Covidence software (https://www.covidence.org/, accessed on 10 February 2026) for screening. The database-specific search strategies for all five databases are presented in Appendix A.

### 2.3. Selecting Studies

Inclusion and exclusion criteria were developed using the “PCC—Population, Context, and Concept” model for this scoping review.

P—Any population ranging from children to older adults.

C—Globally.

C—Integration of oral health into general health, utilizing the oral health workforce.

Detailed inclusion and exclusion criteria are explained in Table 1.

Three researchers, MD, HX and RK, independently screened titles and abstracts against the eligibility criteria. In cases of conflict, consensus was reached through discussion, and MD, RK, and HX then assessed the full texts of the included studies to determine eligibility for the review. Any conflicts were resolved through discussions and adjudication by PB and DH.

**Figure 1:**
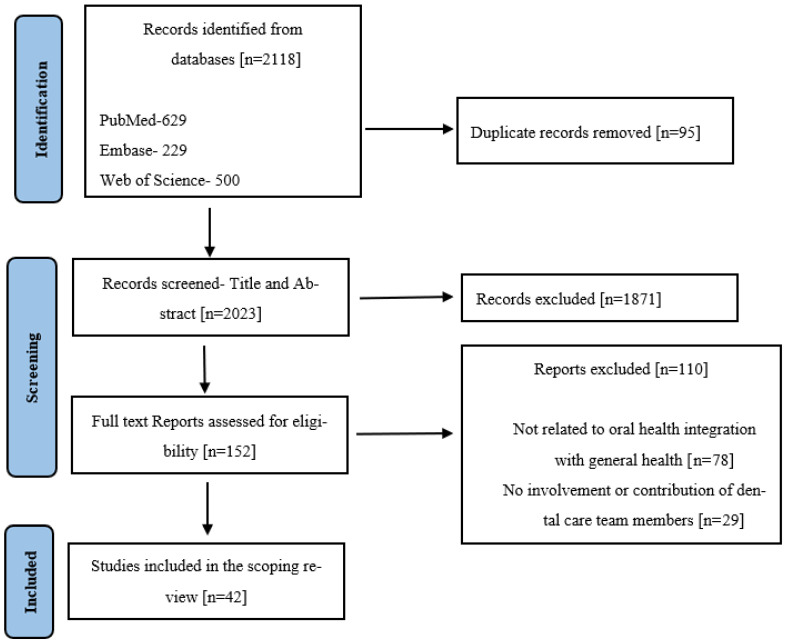
PRISMA diagram for the scoping review.

Details of the number of studies included in the study are presented in a PRISMA flowchart (Figure 1).

### 2.4. Charting Data

Data was extracted using an Excel-based form by MD. This form captured details such as the study title, authors, journal name, aim, study design, country, integration strategy, advantages, disadvantages, facilitators, barriers, and policy implications. During the data charting stage, the study applied the Rainbow Model [17] to categorize the advantages, disadvantages, barriers, and facilitators into three levels: micro, meso, and macro.

### 2.5. Data Analysis and Reporting Results

Content analysis was employed to analyze the data. In this process, all relevant sentences extracted from the included studies were reviewed several times, and initial codes were assigned with suitable labels. These initial codes were subsequently reviewed and refined to attain a more comprehensive meaning, ultimately labeled as the final codes. After a thorough examination of the content represented by these final codes, the main themes and subthemes were formulated. This stage entailed the collective input of all team members to collaboratively shape and refine these themes. Finally, the results were summarized and qualitatively presented in alignment with the developed themes and subthemes.

## 3. Results

Based on the present results, 42 full texts were included for analysis from the initial pool of 2118 retrieved articles. Figure 1 illustrates the systematic research process, encompassing retrieval, screening, and inclusion of articles via PRISMA flowchart (Figure 1).

Of the 42 articles included in the final review, most were conducted in high-income countries, with the largest proportion undertaken in the United States, followed by the Netherlands, the United Kingdom, Australia, Canada, and China. Four studies involved cross-national comparisons or implementation projects spanning multiple countries.

Study designs were diverse and, in some cases, overlapped within individual studies. Qualitative approaches were most common (n = 22), including interviews, focus groups, case studies, and program or implementation evaluations. Quantitative survey studies (n = 12), mixed methods design (n = 8), and retrospective or observational analyses (n = 6) were also represented.

The included studies involved a wide range of stakeholders, including dentists and oral health professionals (n = 30), primary care providers and nurses (n = 21), health service administrators or policymakers (n = 14), insurers or payers (n = 6), and patients or community members (n = 12). Several studies focused specifically on Indigenous populations (n = 5) and older adults (n = 8). These categories were not mutually exclusive, and individual studies often included multiple participant groups.

Across the included articles, multiple integration approaches were examined, most commonly medical–dental collaboration (n = 28), chairside screening for chronic diseases (n = 17), bi-directional referral pathways (n = 15), shared or interoperable electronic health records (n = 13), interprofessional education initiatives (n = 11), and telehealth-based models (n = 10). These approaches frequently co-occurred within studies.

Detailed characteristics of the included studies are presented in Table 2.

The synthesis identified five major integration domains that illustrate how oral health can be systematically integrated into primary and general healthcare through the utilization of oral healthcare professionals (Table 3). Across all included studies, the overarching aim was to improve population health outcomes by embedding oral health within broader health systems and service delivery models. Five principal integration domains were identified in which oral health professionals can play a substantive role within general health settings: [1] chronic disease management, encompassing screening, counseling, and referral; [2] emergency care management; [3] integration of electronic health records (EHRs); [4] interprofessional education across undergraduate, postgraduate, and continuing professional development levels; and [5] telehealth, including tele-dentistry, which emerged as a distinct integration strategy following detailed analysis of study characteristics.

Chronic disease management was the most extensively examined integration domain. The included studies demonstrated that dental professionals could contribute meaningfully to the early identification and management of systemic conditions through screening, counseling, and the establishment of bi-directional referral pathways. At the macro level, facilitators included increased investment and policy support for integrating dental teams, while key barriers related to fragmented governance, the low prioritization of oral health, lack of political leadership, financial and reimbursement constraints, and scope-of-practice and liability concerns. At the meso level, organizational facilitators included the diagnostic potential of dental settings, the use of salivary biomarkers, interprofessional collaboration, and leadership at the service level, whereas weak referral systems, limited interoperability, evidence gaps, and infrastructure costs constrained implementation. At the micro level, regular patient contact with dental services, trust relationships, and professional willingness facilitated integration, while time constraints, role ambiguity, and insurance-related barriers posed challenges.

Emergency management emerged as a smaller but distinct integration domain, highlighting the potential contribution of dental professionals during public health emergencies and disaster situations. The included studies identified scenarios such as mass casualty events, pandemics, bioterrorism preparedness, infectious disease outbreaks, and surge capacity situations in which dental clinics and personnel could be mobilized to support triage, screening, surveillance, infection control, and continuity of essential health services. Well-equipped dental clinics were identified as potential alternative care sites during emergencies at the meso level, while dental professionals’ transferable clinical skills, including infection control expertise, minor surgical competence, and diagnostic capability, were recognized as micro-level facilitators. However, at the macro level, the absence of formal emergency preparedness policies that explicitly integrate dental services into health system response frameworks limited systematic utilization of this workforce capacity.

Electronic health records were identified as a critical enabler of oral and general health integration. Combined dental and medical data were shown to improve risk prediction and care coordination; however, limited interoperability between dental and medical systems, uneven digital infrastructure, governance fragmentation, and patient privacy concerns represented persistent barriers. At the professional level, challenges related to information overload and cross-disciplinary data interpretation were also reported.

Interprofessional education was recognized as foundational to sustainable integration. Positive professional attitudes toward additional training were reported, particularly among dental practitioners; however, gaps remained at the meso and macro levels due to the lack of tailored, practice-relevant curricula and insufficient policy mandates supporting interprofessional education across undergraduate, postgraduate, and continuing professional development pathways.

Finally, telehealth and tele-dentistry emerged as a novel and distinct integration strategy. Studies demonstrated that tele-dentistry could facilitate follow-up, improve coordination between dental and medical providers, and enhance access to care, particularly for underserved populations. At the macro level, alignment with value-based care models supported scalability, while at the meso and micro levels, strengthened professional relationships and improved continuity of care were key advantages.

## 4. Discussion

As a scoping review, this study aims to map the breadth and nature of available evidence rather than assess the quality or effectiveness of interventions; therefore, the findings should be interpreted with appropriate caution given the heterogeneity of included study designs.

This scoping review synthesizes and maps evidence across five integration domains operating at macro, meso, and micro levels, highlighting how oral healthcare professionals may contribute to embedding oral health within broader healthcare systems. Building on these findings, four cross-cutting strategic directions are suggested as potential enablers of system-level integration: (1) explicit policy prioritization and governance reform at the macro level; (2) workforce empowerment and service redesign at the meso level; (3) strengthening digital and referral infrastructure to support coordinated care; and (4) enhancing community literacy, advocacy, and behavioral engagement at the micro level. These strategic directions represent an interpretive synthesis derived from patterns across the five domains rather than additional thematic findings. Collectively, these findings highlight the potential importance of coordinated policy, organizational, and workforce action to support the integration of oral health within general healthcare delivery.

Regarding the integration of oral healthcare services, Christian et al. [14] have summarized several strategies in their scoping review, including the provision of basic oral healthcare services by physicians, emergency department staff, and primary healthcare personnel; training general health professional, nurses, and midwives to provide oral healthcare services; and strengthening collaborations between healthcare workers and dental hygienists/dental therapists. While previous reviews have outlined a range of strategies for strengthening collaboration between dental and non-dental health professionals, including training primary care providers and expanding preventive roles, the findings of this scoping review extend this discussion by identifying specific system-level contexts in which such training may be operationalized. Our findings contribute to the understanding of potential structural and organizational conditions under which workforce strengthening efforts could be embedded within routine healthcare delivery systems, particularly through policy alignment, digital integration, and coordinated referral infrastructures.

First, the findings highlight the need for a paradigm shift in dental education, encompassing both curriculum content and pedagogical approaches. This includes strengthening interprofessional education across undergraduates and postgraduates, and continuing professional development levels to better prepare the oral health workforce for collaborative practice. This direction aligns directly with the integration domains identified in this review, particularly interprofessional education and chronic disease management, where competency gaps and discipline-oriented training structures were identified as key meso-level barriers.

In addition, the findings underscore the importance of embedding dental professionals within coordinated referral and digital information systems to operationalize collaborative practice. Strengthening bi-directional referral networks and improving interoperability between dental and medical information systems may enhance role clarity, continuity of care, and early detection of systemic conditions, facilitators consistently identified across the chronic disease and EHR integration domains.

This transformative approach is supported by broader evidence and aligns with the facilitators identified in this scoping review, including collaborative workforce models and preventive service integration. For example, Nakre et al. [58] demonstrated that oral health promotion programs are generally more effective than education alone, highlighting the value of embedding oral health within broader preventive and interprofessional healthcare frameworks. However, consistent with the barriers identified in this review, implementation of such multidisciplinary approaches requires workforce training, coordination across professional groups, and system-level infrastructure and policy support.

Regarding workforce empowerment and service redesign at the meso level, this review highlights the importance of structurally embedding oral health professionals within healthcare delivery systems. Across multiple integration domains, organizational facilitators included interprofessional collaboration, local leadership, expanded scope-of-practice frameworks, and improved referral coordination. Conversely, discipline-oriented training models, unclear role delineation, and fragmented service structures were recurrent barriers.

While previous reviews have emphasized financial and policy levers to support integration [14], and others have primarily centered on funding mechanisms, cost, and service coverage as dominant drivers of reform [59], the present synthesis underscores the operational importance of organizational redesign and workforce utilization. Positioning oral health professionals as routine contributors to chronic disease management, preventive care, emergency preparedness, and digital information systems moves beyond funding reform alone and reflects a structural reconfiguration of service delivery models. Rather than treating oral health integration as an adjunct initiative, the findings support its conceptualization as standard practice within comprehensive health system design, requiring sustained political commitment and structural inclusion in policy agendas.

At the micro level, enhancing public literacy and community engagement emerged as an important enabler of integration. The findings indicate that both professional attitudes toward expanded roles and patient health-seeking behaviors influence the feasibility of integrated care. Improving oral health literacy may encourage regular preventive visits, strengthen referral adherence, and support shared chronic disease management pathways. Importantly, integration efforts that incorporate community-based prevention and health promotion strategies may strengthen behavioral change beyond clinical service delivery alone. Consistent with the previous literature [60], embedding oral health within broader preventive education and community health initiatives may support sustainable integration, particularly when aligned with interprofessional and system-level reforms identified in this review.

The findings identified persistent barriers across all four strategic directions. At the system level, reimbursement challenges, fragmented financing structures, and limited Medicare or insurance coverage constrained sustainability. At the provider level, referral inefficiencies, workload pressures, part-time workforce participation, and medico-legal concerns limited implementation. Educational barriers included insufficient interprofessional training and limited awareness of screening tools. Digital integration remained constrained by interoperability limitations and concerns regarding data privacy. These findings reinforce the importance of tailoring integration strategies to local system conditions and addressing structural, organizational, and behavioral barriers concurrently to support sustainable implementation.

Despite the strengths of this review, an important limitation should be acknowledged: First, most of the included studies were conducted in the United States, which may limit the generalizability of the findings to other healthcare systems and contexts. Second, the inclusion of diverse study designs, including qualitative studies, surveys, implementation projects, reviews, and commentaries, introduces methodological heterogeneity. As a result, this scoping review does not assess the quality or strength of evidence, and the findings should be interpreted as indicative of emerging patterns and conceptual insights rather than definitive or causal conclusions regarding effectiveness. 

Finally, variations in study contexts, populations, and healthcare systems may influence the transferability of the identified integration strategies, highlighting the importance of adapting findings to local policy, organizational, and service delivery conditions.

## 5. Conclusions

This scoping review synthesizes five integration domains and four cross-cutting strategic directions for embedding oral health within broader healthcare systems. By conceptualizing integration across macro, meso, and micro levels, the study provides a structured framework that may serve as a reference for policymakers, educators, and health service leaders. The findings highlight potential enablers, such as coordinated governance, workforce development, digital infrastructure, and community engagement, which could support integration. Adapting these enablers to local health system contexts will be important for informing future research and exploratory efforts toward sustainable and equitable integration pathways.

## Figures and Tables

**Table 1:** Inclusion and exclusion criteria.

Criteria	Inclusion Criteria	Exclusion Criteria
Study design	All study designs, from observational studies to experimental studies. Original research, reviews, editorials, commentaries.	Book chapters
Study setting	Any study setting, including a clinical setting, a community setting, a school setting, etc.	
Study population	Any study population including children, adolescents, adults, older adults, pregnant mothers, etc.	
Study concept	Integrating oral health into general health through the oral healthcare workforce.	Integrating oral health into general health through other healthcare professionals [non-dental]
Language and period	English 1 January 2000 to 31 December 2024	

**Table 2:** General characteristics of the selected papers.

#	Author’s Name and Year	Journal	Country	Study Design	Aim of the Study	Integration Strategy
1.	Acharya et al., 2018 [18]	*JDR Clinical & Translational Research*	USA	Observational study	To investigate dentists’ ability to contribute to the long-term goal of positively affecting the care of dental patients with dysglycemia.	Integrated dental–medical electronic health record [EHRs].
2.	Atchison et al., 2018 [11]	*Journal of American Dental Association*	USA	Environmental scan	To describe the types of integration efforts that medical and dental primary care delivery systems are embracing, and 4 major types of medical–dental integration through the case studies.	Preventive [non-dental] health services provided by a dental provider in a dental clinic setting.
3.	Blue & Riggs, 2016 [19]	*J Evid Base Dent Pract*	USA	Qualitative study	To describe the potential of an interdisciplinary team-based approach to individual and population health, including oral health, via an accountable care community.	A dental hygienist/therapist in the Accountable Care Organization provides screenings, and advice, and assists in interdisciplinary management.
4.	Cardenas et al., 2023 [20]	*Annal of Family Medicine*	USA, Somaliland, Africa	Project and implementation and evaluation	To improve the early detection of hypertension in the dental setting and of gingivitis in the primary care setting, and to increase the rate of bi-directional referrals between oral and primary care partners.	Teaming and Integrating for Smiles and Health [TISH] Learning Collaborative [medical and dental teams working together to implement screening and referral processes].
5.	Laniado et al., 2021 [21]	*Oral health and preventive dentistry*	USA	Web-based survey	To assess the knowledge and attitudes of dentists at the largest municipal healthcare system in the United States about point-of-care chairside diabetes testing in the dental clinic.	Chairside diabetes testing by dental care providers.
6.	Linabarger et al., 2021 [9]	*Preventing chronic disease, public health research, practice, and policy*	USA	Both qualitative and quantitative surveys	To facilitate, strengthen, and increase collaboration between oral health and chronic disease programs at the state health department around common risk factors for oral health and chronic disease; build synergy; and maximize resources to improve oral health and decrease associated comorbid chronic diseases.	Dental clinicians incorporated in screening, referral, and counseling.
7.	Acharya et al., 2011 [22]	*European Federation for Medical Informatics*	USA	Online survey	To develop an initial understanding of the medical providers’ core dental information needs and opinion of integrated medical–dental electronic health record [iEHR] environment in their workflow.	Integrated medical–dental electronic health record [iEHRs].
8.	Boynes et al., 2017 [23]	*Journal of Rural Health*	USA	Cross-sectional survey	To identify and quantify oral health interprofessional collaborative practice [IPP].	Electronic health record.
9.	Shelley et al., 2012 [24]	*Nicotine & Tobacco Research*	USA	Semi-structured interviews	To interview dental insurers to assess attitudes toward tobacco use treatment in dental practice, pros and cons of offering dental provider reimbursement, and barriers to instituting a tobacco use treatment-related payment policy for dental providers.	Dental clinicians in tobacco use treatment.
10.	Li et al., 2022 [25]	*Frontiers in digital health*	USA	Retrospective, cross-sectional study	To determine [1] the reasons for initiating medical consultations, [2] the information dental providers requested, [3] the information the consulting physician shared in the returned medical consultants, and [4] the time taken to complete the medical consultants.	Collaborative teamwork, multidisciplinary approach, engage in shared care planning and communication systems, reinforcing continuity of care.
11.	Pawloski et al., 2022 [26]	*Health Promotion Practice in Rural Setting*	USA	Descriptive qualitative study	To elicit perspectives and experiences of providers and administrators involved in the MDI program and to assess the acceptability, feasibility, and success of an MDI strategy.	Dental professionals’ role in preventive care with a multidisciplinary approach.
12.	Reynolds et al., 2022 [27]	*Journal of Public Health Dentistry*	USA	Qualitative study	This article presents the results of the second phase of this project, which determined the components, or conceptual subdivisions, associated with each PCDH characteristic.	Collaborative teamwork, interprofessional integration, and oral healthcare team as not only treating disease but also focusing on prevention, patient engagement, care coordination, and chronic disease management in partnership with other providers.
13.	Giddon & Assael, 2018 [28]	*Preventive Medicine*	USA	Review/ Discussion	To assess policy shortcomings and propose strategies to increase organized dentistry’s involvement in integrating oral and general healthcare.	Dentistry needs to reaffirm its dedication to being a healthcare profession, prioritizing health-related aspects over business considerations.
14.	Hilton, 2014 [29]	*Journal of the California Dental Association*	USA	Review	To examine past efforts and identify successes, challenges, and best practices that can strengthen endeavors in all dental practice settings regarding the integration of dental and medical care.	Mixed pack visits, Health Information Technology system integration, and screening and referring patients with chronic diseases to medical clinics.
15.	Janssen & Lampiris, 2007 [30]	*Dent Clin N Am*	USA	Review	To understand the role dentists and dental hygienists can have in emergency response.	Oral health professionals are actively involved in emergency response by raising awareness, building needed partnerships, identifying and garnering resources, facilitating training, policy development, surveillance, and evaluation.
16.	Mann, 2009 [31]	*New York State Dental Journal*	USA	Review	To address the significance of providing patients with comprehensive treatment, prioritizing their overall health needs on par with their oral health.	Dental education [increasing awareness, forging essential partnerships, identifying and securing resources, and facilitating training, policy development, surveillance, and evaluation].
17.	Myers-Wright & Lamster, 2016 [32]	*J Evid Base Dent Pract*	USA	Review	Suggesting that oral healthcare professionals who broaden their scope-of-practice include health promotion strategies and primary health screenings will contribute to reducing risks.	Interprofessional education, common risk factor approach and health promotion utilizing dentists for screening, and electronic health record integration.
18.	Rice, 2021 [33]	*Front. Oral. Health*	USA	Review/perspective piece	To provide examples of the need to bring dentistry into the fold of interdisciplinary approaches in healthcare.	Dental professionals integrated in Alzheimer’s prevention.
19.	Shimpi et al., 2022 [34]	*AMA Journal of Ethics*	USA	Review	To outline the necessity for enhanced interoperability of EHR and discuss how the transfer of health information within the integrated medical and dental practice of the Marshfield Clinic Health System can enhance diabetes care.	EHR integration.
20.	Kopycka-Kedzierawski et al., 2018 [35]	*Telehealth*	USA	Review	To describe the advancement and uses of tele-dentistry at the University of Rochester’s Eastman Institute for Oral Health [EIOH] as an integral component of the oral healthcare system and its relation to the general telehealth initiative within the university’s Medical Center as a whole.	Telehealth and tele-dentistry.
21.	Gauger T. L. et al., 2018 [36]	*Journal of Public Health Dentistry*	USA	Scoping review	To present and evaluate different types of care models that exist between oral health and primary care providers in pediatric settings.	Integrated EHR shared by the medical and oral health professionals, a referral system, and interprofessional education.
22.	Sparer, 2011 [37]	*American Journal of Public Health*	USA	Commentary	To discuss the Patient Protection and Affordable Care Act; where does the oral health community fit in this initiative?	Dentists are an important component in primary healthcare.
23.	Adesanya et al., 2016 [38]	*Public Health Reports*	USA	Reports and recommendations	To discuss the Oral Health Strategic Framework, 2014–2017.	Electronic health record.
24.	Fellows et al., 2022 [39]	*Journal of the American Dental Association*	USA	Cover story	To highlight key advances and continuing challenges regarding oral health status, access to care and the delivery system, integration of oral and systemic health, financing of oral healthcare, and the oral health workforce.	Fully integrated electronic health records.
25.	Riddle, 2020 [40]	*Oral Diseases*	USA	Proceeding	To discuss the approaches to ending the HIV epidemic.	HIV screening.
26.	Dolce et al., 2016 [41]	*Journal of Interprofessional Care*	USA	Guest Editorial	To outline how oral health competencies can be used by educators across all health professions to advance interprofessional education.	Interprofessional education.
27.	Robinson, 2016 [42]	*Journal of the California Dental Association*	USA	Editorial	To identify key drivers and challenges, examine opportunities for greater interprofessional collaboration, and create a vision for the dentist of the future.	Interprofessional education and practice.
28.	Niesten et al., 2021, part 1 [43]	*Gerodontology*	Netherlands	Qualitative study	To synthesize a framework of barriers and facilitators in the normative integration of oral healthcare [OHC] into general healthcare for frail older adults at macro [system], meso [organization and interprofessional integration], and micro [clinical practice] levels.	Oral health teams need to be structurally included in healthcare delivery [e.g., policies, financial incentives, and organizational support].
29.	Niesten et al., 2021, part 2 [44]	*Gerodontology*	Netherlands	Qualitative study	To synthesize a framework of barriers and facilitators in the functional integration of oral healthcare [OHC] into general healthcare for frail older adults at macro [system], meso [organization and interprofessional integration], and micro [clinical practice] levels.	Interprofessional collaboration and structural inclusion of oral health in healthcare systems [e.g., funding, policy frameworks].
30.	Everaars et al., 2018 [45]	*The University of Manchester Research*	UK and the Netherlands	Qualitative interviews and discussions	To compare the views from the two countries on the future priorities for service provision, and to discuss these results in the context of a quality framework for older people.	Dental professionals’ role in preventive care with a multidisciplinary approach.
31.	Kossioni A E, 2012 [46]	*Gerodontology*	Europe	Short report	To discuss the preparedness of the social and healthcare systems and the health workforce in Europe to manage the increasing general and oral healthcare needs of older adults.	Multidisciplinary approach.
32.	Yun et al., 2022 [47]	*Healthcare*	China and Australia	A quantitative online survey	To explore the knowledge, experience, and perspectives of Chinese community medical practitioners, dentists, and community nurses on the management of diabetes and periodontitis and interprofessional collaboration in the primary healthcare setting in China.	Management of diabetes and periodontitis and interprofessional collaboration.
33.	Chan et al., 2023 [48]	*Geriatrics*	China	Communication	To discuss the merits, outline the challenges, and propose approaches to integrating oral health into general health services for older adults.	Promoting primary healthcare by controlling shared risk factors by the dental team.
34.	Ghorbani et al., 2018 [49]	*Eastern Mediterranean Health Journal*	Iran	Qualitative research	To examine problems in integrating oral health services into PHC.	Collaborative teamwork, highlighting the role of the dental team in developing guidelines, providing training, supervising, and managing referrals within the integrated model.
35.	Shrivastava et al., 2019 [50]	*BMJ OPEN*	Canada	Qualitative approach and developmental evaluation methodology	This study explored the patients’ perspectives of patient-centered integrated care in oral health services integrated into a primary healthcare organization serving a northern Quebec Cree population.	Collaborative teamwork; oral health team is included in planning and policy frameworks.
36.	Antonarakis et al., 2011 [51]	*Health Promotion Practice*	Guatemala	Review	To describe how promoting dental health is integrated into family-oriented health promotion approaches in Guatemala.	Collaborative teamwork, or health professionals contributing to prevention programs, which help embed oral health into chronic disease management.
37.	Lin et al., 2023 [52]	*Health Research Policy and Systems*	Malaysia	Study Protocol	To assess the influence of dental workforce training and education programs on policy evolution in Malaysia.	Interprofessional education.
38.	Varenne, 2015 [53]	*Journal of Dental Education*	WHO African region	WHO meeting summary	To provide an overview of the WHO strategic initiatives regarding oral health in the WHO African region.	Integrated disease prevention and oral health promotion and surveillance system.
39.	Harnagea et al., 2017 [3]	*BMJ*	Global	Scoping review	To map the literature and provide a descriptive synthesis on the barriers and facilitators of the integration of oral health into primary care.	Interdisciplinary primary care, managing shared risk factors for chronic conditions [e.g., diabetes, cardiovascular disease].
40.	Lee et al., 2018 [54]	*J Public Health Pol*	Global	Viewpoint	To highlight oral health from a global health perspective, calling for all public health leaders to advocate for the oral health of all.	Actors in the prevention and management of non-communicable diseases; dentists contribute alongside other providers in tackling tobacco use, poor diet, alcohol consumption, and hygiene. Capacity building.
41.	Bowen, 2016 [55]	*The Journal of Dental Hygiene*	N/A	Commentary	To discuss the dental hygienist’s role in dental medical integration.	Dental hygienist acting as an oral healthcare manager.
42.	Sheiham, 1992 [56]	*International Dental Journal*	N/A	Review	To discuss the role of the dental team in chronic disease management.	Health promotion through common risk factor approach.

**Table 3:** Integrating oral health into primary/general health through utilization of oral healthcare professionals.

Domain 1. Chronic Disease Management [Screening, Counseling, Referral]			
Level	Facilitators	Barriers	Advantages/Disadvantages
**Macro [system/policy]**	Increased investment in integrating dental teams, offset by improved chronic disease outcomes [17]; financial support from governments and non-profit organizations [3].	Oral health treated as a standalone service [25,49,51,54]; low policy priority for oral health [17,56]; lack of political leadership and enabling policies [3,46,49,54]; absence of established referral and back-referral networks [8]; lack of financial sustainability and reimbursement mechanisms, including separate billing systems, lack of diagnostic codes, and limited Medicare/insurance coverage [8,17,18,19,21,47,57]; scope-of-practice and liability constraints [39,49]; long waiting lists [50].	Cost-effective training of dental auxiliaries and educators [32]; more efficient use of health system resources.
**Meso [organizational/service]**	Use of routine dental investigations [e.g., panoramic radiographs] to detect systemic risks such as carotid atherosclerosis [31]; salivary biomarkers for systemic disease identification [55]; dental teams as key players and catalysts in chronic disease detection and management [38,40,43,44,54]; interprofessional education, role clarity, co-location, case management, and local strategic leadership [3]; existing organizational networks [51].	Limited research outcomes and evidence base [17,18]; lack of access to medical and dental providers for referral and back-referral [8,43,44,49]; weak or poorly functioning referral systems [3]; lack of communication between services [43,44]; cost of equipment, supplies, and administrative infrastructure [19].	Early identification of new or poorly managed conditions [17,18]; improved and timely referrals to medical services [17,18]; enhanced communication and comprehensive care planning [39,56]; shared organizational resources [56]; use of dental auxiliaries as an affordable workforce option [51]
**Domain 1. Chronic disease management [screening, counseling, referral] continued**			
**Level**	**Facilitators**	**Barriers**	**Advantages/Disadvantages**
**Micro—Patients**	Regular attendance at dental appointments [3]; holistic and welcoming dental environments providing psychological support [22,50].	Resistance to receiving medical services from dental teams [19].	Timely interdisciplinary management reduces complications and improves quality of life [17,18]; expanded access to care, particularly in health-professional-shortage areas [36]; continuity and quality of care [25,27,50]; team-based care and avoidance of adverse events [25,43,44,45].
**Micro—Dental care professionals**	Willingness to provide screening and preventive services [19,40]; existing clinical knowledge and skills [20,55]; strong trusting relationships with patients [31]; professional identity as integrated care providers [26,45].	Lack of awareness of screening tools [e.g., AUSDRISK] [20]; insufficient training in health advice and geriatric medicine [46,51,54]; limited time, part-time availability, and workload constraints [56]; reluctance to treat uninsured patients [8]; concerns about legal consequences and liability [49]; lower affinity for geriatric integration roles [45].	Increased professional value, flexibility, and engagement in integrated care models [26,45].
**Micro—Medical professionals**	Positive perceptions of dental teams’ role in screening and referral [17,20]; easier access to patient information through integration [22].	High workload and time constraints [56]; reluctance to see uninsured patients [8]; lack of competencies and awareness regarding oral–systemic links [3,43,44,45]; lack of incentives [49].	Reduced screening burden as chronic disease prevalence increases [30]; clearer role distribution and shared responsibility [49].
**Domain 2. Emergency management**			
**Level**	**Facilitators**	**Barriers**	**Advantages/Disadvantages**
**Macro**	—	Absence of formal emergency preparedness policies integrating dental services.	Underutilization of dental workforce capacity.
**Meso**	Well-equipped dental settings capable of supporting emergency response [28].	—	Potential alternative sites of care during emergencies.
**Micro—Dental care professionals**	Strong sense of professional responsibility during disasters; substantial transferable clinical experience [28].	—	Augments surge capacity and continuity of essential services.
**Domain 3. Electronic health records [EHRs]**			
**Level**	**Facilitators**	**Barriers**	**Advantages/Disadvantages**
**Macro**	—	Maldistribution of digital resources and infrastructure [23].	—
**Meso**	—	Lack of interoperability between dental and medical information systems [47].	Improved accuracy of risk prediction when dental and medical data are combined [14].
**Micro—Patients**	—	Privacy concerns and information overload related to shared records [22,55].	Improved clinical outcomes and reduced time for urgent care [26].
**Micro—Dental and Medical professionals**	Easier access to patient information [22].	Challenges in interpreting dental or medical jargon [22].	Reduced duplication of work and improved efficiency [26].
**Domain 4. Interprofessional education [undergraduate, postgraduate, and continuing professional development]**			
**Level**	**Facilitators**	**Barriers**	**Advantages/Disadvantages**
**Macro**	Integration of oral health as a vehicle for advancing interprofessional education [41].	Inadequate cross-disciplinary training mandates [39].	System-wide culture changes toward collaboration.
**Meso**	—	Lack of appropriate and tailored interprofessional curricula [47].	—
**Micro—Dental care professionals**	Positive attitudes toward additional training [28].	Overloaded curricula and competing educational demands [41].	Improved role clarity and collaborative competence.
**Domain 5. Telehealth and tele-dentistry**			
**Level**	**Facilitators**	**Barriers**	**Advantages/Disadvantages**
**Macro**	Alignment with value-based care models [35].	—	Supports system-level integration and scalability.
**Meso**	Tele-dentistry facilitating follow-up and coordination of care [35].	—	Improves continuity of care.
**Micro—Patients and professionals**	Strengthened professional relationships and access to specialist input [35].	—	Improved access to quality care, particularly for underserved populations.

## Data Availability

No new data were created or analyzed in this study. Data sharing is not applicable to this article.

## Appendix A

### Database-Specific Search Strategies

*PubMed-629*

*((((“Oral Health”[MeSH Terms] AND “Public Health”[MeSH Terms]) OR “Primary Health Care”[MeSH Terms] OR “Patient-Centered Care”[MeSH Terms] OR ((“collaborate”[All Fields] OR “collaborated”[All Fields] OR “collaborates”[All Fields] OR “collaborating”[All Fields] OR “collaboration”[All Fields] OR “collaborations”[All Fields] OR “collaborative”[All Fields] OR “collaborative s”[All Fields] OR “collaboratively”[All Fields] OR “collaboratives”[All Fields] OR “collaborator”[All Fields] OR “collaborators”[All Fields]) AND (“practicability”[All Fields] OR “practicable”[All Fields] OR “practical”[All Fields] OR “practicalities”[All Fields] OR “practicality”[All Fields] OR “practically”[All Fields] OR “practicals”[All Fields] OR “practice”[All Fields] OR “practices”[All Fields] OR “practiced”[All Fields] OR “practices”[All Fields] OR “practicing”[All Fields]))) AND “Systems Integration”[MeSH Terms]) OR “Community Integration”[MeSH Terms])*

*Embase-229*

*((((‘Oral Health’/exp AND ‘Public Health’/exp) OR ‘Primary Health Care’/exp OR ‘Patient-Centered Care’/exp OR ((collaborate OR collaborated OR collaborates OR collaborating OR collaboration OR collaborations OR collaborative OR ‘collaborative s’ OR collaboratively OR collaboratives OR collaborator OR collaborators) AND (practicability OR practicable OR practical OR practicalities OR practicality OR practically OR practicals OR practice OR ‘practice s’ OR practiced OR practices OR practicing))) AND ‘Systems Integration’/exp) OR ‘Community Integra-tion’/exp)*

*Web of Science-500*

*((((“Oral Health” AND “Public Health”) OR “Primary Health Care” OR “Patient-Centered Care” OR ((collaborate OR collaborated OR collaborates OR collaborating OR collaboration OR collaborations OR collaborative OR “collaborative s” OR collaboratively OR collaboratives OR collaborator OR collaborators) AND (practicability OR practicable OR practical OR practicalities OR practicality OR practically OR practicals OR practice OR “practice s” OR practiced OR practices OR practicing))) AND “Systems Integration”) OR “Community Integration”)*

*Scopus-714*

*((((“Oral Health” AND “Public Health”) OR “Primary Health Care” OR “Patient-Centered Care” OR ((collaborate OR collaborated OR collaborates OR collaborating OR collaboration OR collaborations OR collaborative OR “collaborative s” OR collaboratively OR collaboratives OR collaborator OR collaborators) AND (practicability OR practicable OR practical OR practicalities OR practicality OR practically OR practicals OR practice OR “practice s” OR practiced OR practices OR practicing))) AND “Systems Integration”) OR “Community Integration”)*

*ProQuest-46*

*((((MESH.EXACT.EXPLODE(“Oral Health”) AND MESH.EXACT.EXPLODE(“Public Health”)) OR MESH.EXACT.EXPLODE(“Primary Health Care”) OR MESH.EXACT.EXPLODE(“Patient-Centered Care”) OR ((NOFT(collaborate) OR NOFT(collaborated) OR NOFT(collaborates) OR NOFT(collaborating) OR NOFT(collaboration) OR NOFT(collaborations) OR NOFT(collaborative) OR NOFT(“collaborative s”) OR NOFT(collaboratively) OR NOFT(collaboratives) OR NOFT(collaborator) OR NOFT(collaborators)) AND (NOFT(practicability) OR NOFT(practicable) OR NOFT(practical) OR NOFT(practicalities) OR NOFT(practi-cality) OR NOFT(practically) OR NOFT(practicals) OR NOFT(practice) OR NOFT(“practice s”) OR NOFT(practiced) OR NOFT(practices) OR NOFT(practicing)))) AND MESH.EXACT.EXPLODE(“Systems Integration”)) OR MESH.EXACT.EXPLODE(“Community Integration”))*
